# Treatise on Nasal Catarrh and Allied Diseases

**Published:** 1886-07

**Authors:** W. E. Casselberry


A PRACTICAL TREATISE ON NASAL CATARRH AND ALLIED DISEASES. *By* BEVERLY ROBINSON, A. M., M. D. (*Paris*) ; *Clinical Professor of Medicine at the Bellevue Hospital Medical College, New York ; physician to St. Luke's and Charity Hospitals, etc., etc. Second Edition, revised and enlarged, with one hundred and fifty-two wood engravings. Octavo, pp. xii., 276. New York:* WILLIAM WOOD & COMPANY: *Chicago:* W. T. KEENER.

A comparison between the first and second editions of Prof. Robinson's treatise is exceedingly gratifying to the rhinologist. The original issue of 1880 was the first monograph of importance published on the subject in this country. It was especially adapted by its practical character and excellence to supply the urgent desire which had then arisen for a lucid exposition of the subject of nasal catarrh, and it enjoyed, in consequence, an extended popularity to which the second edition, now issued, is well worthy of inheritance.

The author struck the key-note when, in the very first pages, he demonstrated the hydra to be comprised, not of a single affection but of twenty-nine distinct and separate diseases, and as each of the twenty-nine received mostly different and appropriate medication, the riddle of treatment approached solution.

In the new edition, the same division of the subject is retained and then enlarged upon in the subsequent text.

Two beautiful illustrations of the reticular structure of the turbinated bodies convey a clear idea of the special erectile character of this tissue upon which the several varieties of chronic rhinitis, rhinitis simplex, rhinitis hypertrophica and rhinitis atrophica so largely depend. The prophylaxis of "taking cold" is expounded in a clear and forcible manner, particularly as regards the care of the feet, cold sponge bathing, frictions and massage, clothing, temperature and mode of heating living apartments; all measures, "important to the well being of the majority of people" but "absolutely essential to the prophylaxis or cure of those affected with catarrhal inflammations of the nasal fossæ."

The author very properly insists that an acute coryza, in its incipiency, and later if necessary, should be systematically treated, both as a means of immediate relief, and as a prophylactic measure against chronic pathological changes of a catarrhal nature. The universal custom of letting a cold "run its course" is certainly to be deprecated. How frequently do we observe an acute rhinitis, continued thus for weeks, extending to the ears, eyes, larynx and bronchi, perhaps even recurring with augmented severity, again and again throughout the season. Such neglect is the primary cause of many cases of deafness, of chronic laryngitis and pharyngitis with impairment of voice, of follicular disease of the naso-pharyngeal space, and of hypertrophic nasal catarrh, not to mention pulmonary affections, still more serious because of fatal tendencies.

Cocaine is unmentioned in this connection, doubtless because its introduction followed the preparation of the text. A one to two per cent. solution applied in the form of a warm spray is the most effective of local applications. A point well taken, and from which many profit, is that ulceration of the nasal cartilages and bones, even perforation of the septum, should not invariably be adjudged syphilitic. Scrofula, catarrh, febrile diseases, *e.g.*, typhoid fever, measles, etc., certain toxic substances, as arsenic and mercury, are all regarded as causes. We have reason to believe, also, that workers in lead not uncommonly suffer from such extreme forms of atrophic nasal catarrh as to lead to perforation of the septum and ulceration of other parts.

It is impossible to enumerate all the good features of the work. Most of the more recent special surgical procedures are described and the apparatus illustrated.

It is indeed to be regretted that the galvano-cautery, now found so extremely useful in naso-pharyngeal surgery, should not have received more extended attention. The most perfect apparatus of the sort, the Seiler-Flemming battery, remains entirely unnoticed.

The phraseology of the book is clear and concise, the paper and print are fairly good, and typographical errors are few in number.

W. E. CASSELBERRY.

